# Long-Term Kidney and Maternal Outcomes After Pregnancy in Living Kidney Donors

**DOI:** 10.3389/ti.2023.11181

**Published:** 2023-06-28

**Authors:** Marleen C. van Buren, Jildau R. Meinderts, Christiaan A. J. Oudmaijer, Margriet F. C. de Jong, Henk Groen, Tessa Royaards, Louise Maasdam, Mirjam Tielen, Marlies E. J. Reinders, A. Titia Lely, Jacqueline van de Wetering

**Affiliations:** ^1^ Department of Internal Medicine, Erasmus MC Transplant Institute, University Medical Center, Rotterdam, Netherlands; ^2^ Department of Nephrology, University Medical Center Groningen, Groningen, Netherlands; ^3^ Department of Epidemiology, University of Groningen, Groningen, Netherlands; ^4^ Department of Obstetrics, Wilhelmina Children’s Hospital Birth Center, University Medical Center Utrecht, Utrecht, Netherlands

**Keywords:** kidney function, living kidney donation, pregnancy, hypertension, BMI

## Abstract

For counseling it is important to know if pregnancy after Living Kidney Donation (LKD) affects long-term outcomes of the mono-kidney and the mother. Therefore, we performed a retrospective multicenter study in women ≤45 years who donated their kidney between 1981 and 2017. Data was collected via questionnaires and medical records. eGFR of women with post-LKD pregnancies were compared to women with pre-LKD pregnancies or nulliparous. eGFR before and after pregnancy were compared in women with post-LKD pregnancies. Pregnancy outcomes post-LKD were compared with pre-LKD pregnancy outcomes. 234 women (499 pregnancies) were included, of which 20 with pre- and post-LKD pregnancies (68) and 26 with only post-LKD pregnancies (59). Multilevel analysis demonstrated that eGFR was not different between women with and without post-LKD pregnancies (*p* = 0.23). Furthermore, eGFR was not different before and after post-LKD pregnancy (*p* = 0.13). More hypertensive disorders of pregnancy (HDP) occurred in post-LKD pregnancies (*p* = 0.002). Adverse fetal outcomes did not differ. We conclude that, despite a higher incidence of HDP, eGFR was not affected by post-LKD pregnancy. In line with previous studies, we found an increased risk for HDP after LKD without affecting fetal outcome. Therefore, a pregnancy wish alone should not be a reason to exclude women for LKD.

## Graphical Abstract

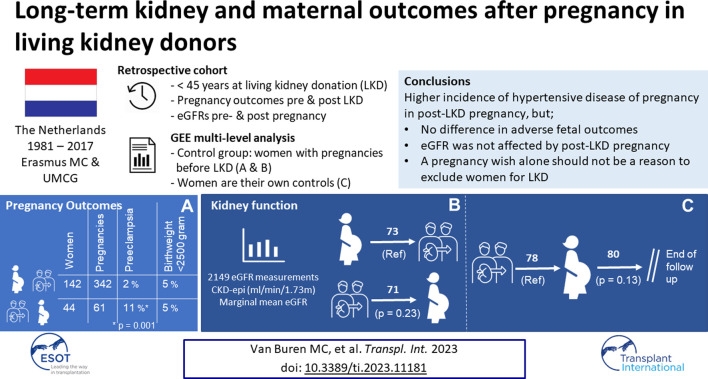

## Introduction

Living donor kidney transplantation is the best treatment option for patients with end-stage renal disease (ESRD), resulting in better outcomes than dialysis as well as deceased donor kidney transplantation. Less is known about the long-term effects of living kidney donation (LKD) on the health of the donor. Existing research is reassuring and shows no increased cardiovascular risk for donors compared to the control group [1]. However, accurate life-time risk assessment of especially young donors, who spend many years with one kidney, remains uncertain. Current literature reports conflicting results on long-term follow-up, predominantly caused by the difficulty of finding a representative control group [2]. A substantial number of donors are women of fertile age and therefore it is of great importance to know if pregnancy affects long-term outcomes and function of the mono-kidney and if LKD affects pregnancy outcomes.

Previous research shows that LKD reduces pre-donation glomerular filtration rate (GFR) by an average of 30% [3]. The remaining kidney experiences compensatory hypertrophy and hyperfiltration and thereby an increase in GFR [4]. A similar increase in GFR is seen during pregnancy, when GFR and renal plasma flow (RPF) increase by 40%–65% and 50%–85% respectively. A pregnancy potentially adds an additional strain of hyperfiltration on the single kidney after LKD [5, 6]. In the general population, pregnancy with reduced GFR due to kidney disease is associated with adverse pregnancy outcomes [7]. Two studies on pregnancy outcomes of women with a single kidney showed an increase in preterm delivery and preeclampsia compared to women with two kidneys [8, 9].

Research is limited on pregnancy outcomes in otherwise healthy living kidney donors, as was concluded by a recent systematic review by Pippias et al. [10]. They reported an increased risk of hypertensive disorders during pregnancy (hypertension and preeclampsia) post-donation, based on four retrospective cohort studies with limited quality. However, they emphasized that the absolute risk of pregnancy-related complications remains very small. Moreover, fetal and neonatal outcomes were not different when comparing pre-donation pregnancies to post-donation pregnancies [10].

To the best of our knowledge, the effect of pregnancy after LKD on eGFR slope has not been investigated yet. Long-term kidney function post-LKD pregnancy has only been reported by Ibrahim et al. [11]. They reported that the serum creatinine of women with post–LKD pregnancies was not different from women with pre-LKD pregnancies using one time measurements at different time points after LKD without information on eGFR slope before and after LKD [11]. It could be possible that the increased strain of pregnancy on the mono-kidney increases the risk of (accelerated) decline of kidney function in the long-term. Therefore, the aim of this research was to assess if long-term kidney function after LKD is prone to a faster decline after pregnancy. Secondly, we assessed if post-LKD pregnancies have a higher risk of complications than pre-LKD pregnancies in our cohort.

## Methods

### Study Design

In this retrospective cohort study, all women who underwent LKD between 1980 and 2017 at the age of 45 years or younger in the University Medical Center Groningen (UMCG) or the Erasmus Medical Center Rotterdam (Erasmus MC) were eligible for inclusion. The study was approved by the medical ethical committees of the UMCG (METc 2014/077) and the Erasmus MC (MEC-2013-585).

### Data Collection and Definitions

LKD-specifics and obstetric characteristics of pregnancies pre-LKD and post-LKD were recorded. Check-ups of eGFR, proteinuria, blood pressure, weight and medication-use are (bi-) annually measured as part of the standard care after LKD in the Netherlands. eGFR was calculated using the CKD-EPI formula [12]. Due to the study design, data on pregnancy outcomes were self-reported by questionnaires sent via post or email. Participants who did not respond were contacted twice by telephone to conduct the questionnaire by a direct interview after obtaining direct informed consent from the subject. When this was not possible, the questionnaire was sent again by post or email. If a donor reported gestational hypertension, preeclampsia and/or Intra Uterine Fetal Demise (IUFD) during post-LKD pregnancy in the questionnaire, their medical record was obtained after written informed consent.

Gestational hypertension was defined as ≥140/90 mmHg measured twice or increase in diastolic blood pressure (DBP) >15 mmHg/systolic blood pressure (SBP) >30 mmHg after 20 weeks of pregnancy, without signs of preeclampsia, according to the guideline of the National Institute for Health and Care Excellence (NICE) [13]. Preeclampsia was defined as it was diagnosed by the attending physician according to the guideline in use, defining preeclampsia as the presence of pregnancy induced hypertension at >20 weeks of gestation and proteinuria [14]. Hypertensive disorders of pregnancy (HDP) were defined as a combined endpoint of either gestational hypertension and/or preeclampsia and/or Hemolysis Elevated Liver enzymes Low Platelets syndrome (HELLP). Miscarriage was defined as the spontaneous loss of a pregnancy before 20 weeks and abortion as the deliberate termination of pregnancy before 20 weeks. IUFD was defined as stillbirth after 20 weeks of pregnancy.

During follow-up, hypertension was defined as a SBP ≥140 mmHg, a DBP ≥90 mmHg and/or the use of antihypertensive medication. Proteinuria was defined as urine protein/creatinine ratio >15 g/mol, urine albumin/creatinine ratio >3.5 mg/mmol or when no ratio could be calculated, proteinuria >0.15 g/L. When none of these quantitative urine measurements were available, a positive urine dipstick was defined as proteinuria.

### Statistical Analyses

Statistical analysis was performed using SPSS software version 25 and GraphPad Prism version 9.3.1. Continuous variables were reported as mean with standard deviation (SD) in case of a normal distribution and as median with interquartile range (IQR) in case of skewed distribution.

#### Effect of Pregnancy on eGFR After LKD

This was analyzed at patient level. Firstly, eGFR slope of women with post-LKD pregnancies were compared with women with only pre-LKD pregnancies or nulliparous. This multivariable analysis was performed using Generalized Estimating Equations (GEE) multilevel analysis and an unstructured correlation matrix was used [15]. The number of months after LKD of each individual measurement was used as the within-subject level and as a continuous covariate (transformed to years after LKD). The two groups were adjusted for differences at baseline. For calculating eGFR slope per year, the interaction term “years after LKD*pregnancy after donation” was used.

Furthermore, a sub-analysis was performed of eGFR slopes before and after pregnancy in women with post-LKD pregnancies (>20 weeks). eGFR measurements within 180 days after LKD were excluded since eGFR post-LKD rises in the first period after LKD [16]. eGFR during pregnancy and in the first month after pregnancy was also excluded, since eGFR is physiologically higher during pregnancy [6]. For calculating eGFR slope per year the interaction term “years after LKD*after first pregnancy” was used. In all analyses, a *p*-value <0.05 was considered statistically significant.

The second part consisted of comparing pregnancy outcomes of pre-LKD pregnancies with pregnancy outcomes of post-LKD pregnancies. Descriptive statistics were used to describe (complicated) pregnancy outcomes. The experimental level in this part of the study were pregnancies instead of women. Descriptive statistics and multi-level analysis with GEE were performed to account for multiple pregnancies in one woman. Analysis was adjusted for differences in baseline between the two groups. An unstructured correlation matrix structure was used for the multilevel univariable analysis, and an exchangeable correlation matrix structure for the multivariable analysis. Odds ratios (OR) were calculated. Predictors that were statistically significant (*p* < 0.05) were added to the multivariable models.

For both parts relevant predictors were selected based on literature: race, parity, age at delivery, age at LKD, BMI before LKD, mean arterial pressure (MAP) before LKD, eGFR before LKD, year of LKD and year of delivery.

## Results

**FIGURE 1:**
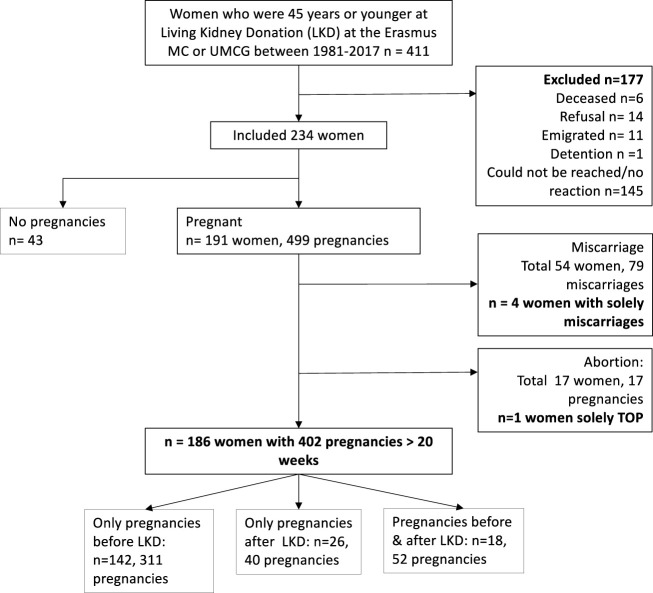
Flowchart.

We included 234 women with 499 pregnancies and 43 nulliparous (Figure 1). Baseline characteristics are shown in Table 1. Most of the pregnancies occurred pre-LKD (75%, *n* = 372). 20 women had pre- and post-LKD pregnancies. Women with post-LKD pregnancies (study group) were younger at LKD then women with only pre-LKD pregnancies (30 years vs. 39 years (*p* < 0.001). eGFR before LKD was significantly higher 118 mL/min/1.73 m^2^ versus 104 mL/min/1.73 m^2^ (*p* < 0.001), respectively. Women with post-LKD pregnancies were older at their first delivery (33 versus 25 years). Years of follow-up after LKD was not significantly different between the two groups (13 vs. 12 years (*p* = 0.28).

**TABLE 1:** Baseline characteristics and pregnancy outcomes per woman after living kidney donation (LKD), *n* = 234 women, 499 pregnancies.

	All	Only pregnancies before LKD	Only pregnancies after LKD	Pre- and post-donation pregnancies	Women after LKD with no pregnancies
Number of donors	234	145	26	20	43
Number of pregnancies	499	372	59	68	N/A
Miscarriage, number (%)	79 (16)	48 (13)	19 (32)	12 (18)	N/A
Abortion, number (%)	17 (3)	11 (<1)	1 (2)	5 (24)	N/A
Pregnancies > 20 weeks					
Number of donors	229	142	26	18	43
Number of pregnancies	402	311	40*	52	N/A
Primipara, number (%)	402	142 (46)	26 (65)	18 (100)	N/A
Total pregnancies per donor, number (%)					
1	48 (26)	33 (23)	15 (58)	0	N/A
2	81 (44)	65 (46)	9 (35)	7 (39)	N/A
>3	57 (31)	44 (31)	2 (8)	11 (61)	N/A
IUFD number (%)	2 (<1)	1 (<1)	1 (2)	0	N/A
Race- White/Caucasian, number (%)	199 (85)	124 (87)	22 (85)	12 (67)	37 (86)
Age at LKD (years), mean (±SD)	37 (9)	40 (4)	30 (5)	31 (4)	37 (7)
Age at first delivery (years), mean (±SD)	26 (5)	25 (5)	33 (5)	24 (4)	N/A
BMI at LKD (kg/m^2^), median {IQR}	25 (I6)	26 (6)	24 (5)	25 (5)	26 (6)
MAP at LKD (mmHg), median {IQR}	90 (11)	90 (13)	91 (8)	89 (11)	91 (9)
Hypertensive drug use at LKD, number (%)	3 (1)	3 (1)	0	0	0
Creatinine at LKD (umol/L), mean (±SD)	61 (10)	68 (14)	68 (13)	60 (18)	66 (11)
eGFR at LKD (ml/min/1.73 m^2^), mean (±SD)	101 (16)	98 (15)	107 (15)	114 (12)	102 (17)

Miscarriage: spontaneous loss of a pregnancy <20 weeks. Abortion: the deliberate termination of pregnancy <20 weeks. IUFD: intra uterine fetal demise >20 weeks of pregnancy, BMI: Body Mass Index, MAP: Mean Arterial Pressure. eGFR (estimated glomerular filtration rate) calculated using the CKD-epi formula. Hypertension defined as systolic blood pressure ≥140 mmHg and or a diastolic blood pressure ≥90 mmHg and or the use of antihypertensive medication. Proteinuria defined as urine protein/creatinine ratio >15 g/mol creatinine, or urine albumin/creatinine ratio >3.5 mg/mmol or when no ratio could be calculated urine proteinuria >0.15 g/L. When none of these quantitative urine measurements were available a positive urine dipstick was defined as proteinuria. *1 woman had no pregnancies >20 weeks before LKD only an abortion. So, when dividing the group of women with pregnancies >20 weeks she moved to the group of only pregnancies after LKD.

### Comparing eGFR Slopes of Women With Post-LKD Pregnancies to Women With Only Pre-LKD Pregnancies or Nulliparous

**FIGURE 2:**
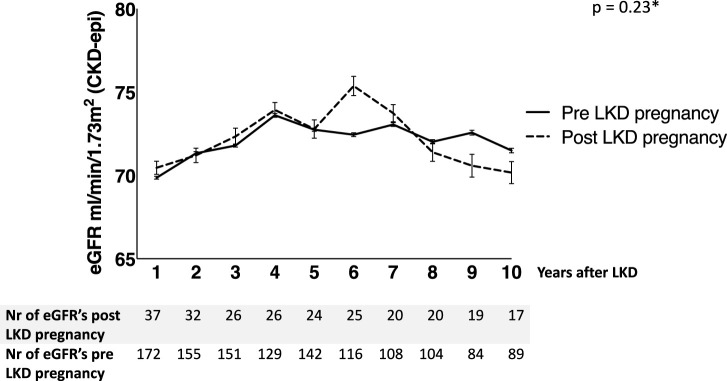
Adjusted mean eGFR after living kidney donation (LKD); women who were pregnant before LKD or nulliparous versus women who got pregnant after LKD (GEE multilevel analysis).

We compared post-LKD eGFR of women with post-LKD pregnancies (study group) to women with only pre-LKD pregnancies or nulliparous (control group). 221 Women were included with 2149 eGFR measurements. Five women were excluded for this analysis because no eGFR levels were available. Multilevel analysis was adjusted for age at LKD, eGFR before LKD, years after LKD and maximum follow-up time. Mean adjusted eGFR in the study group was not significantly different compared to the control group: 71 mL/min/1.73 m^2^ (SEM 1.32, 95% CI: 68.51–73.70) versus 73 mL/min/1.73 m^2^ (SEM 0.57, 95% CI 71.88–74.11, *p* = 0.23). As shown in Figure 2, eGFR increased in both groups the first 4 years after LKD. Adjusted eGFR slope per year in the study group was −0.21 mL/min/1.73 m^2^ per year (SEM 0.09, 95% CI: −0.38 to −0.04) versus −0.15 mL/min/1.73 m^2^ per year (SEM 0.05, 95% CI −0.25 to −0.05) in the control-group (*p* = 0.06). When comparing women with and without HDP pre-LKD, no difference was observed in adjusted mean eGFR after LKD (73 mL/min/1.73 m^2^ (SEM 2.39, 95% CI 68.10–77.46) versus 71 mL/min/1.73 m^2^ (SEM 0.70, 95% CI 69.76–72.51, *p* = 0.51)). Furthermore, there was no difference in eGFR slope in women with and without HDP pre-LKD (−0.29 mL/min/1.73 m^2^ vs. −0.19 mL/1.73 m^2^ (SEM 0.12, 95 CI: −0.33–0.13, *p* = 0.39)).

### eGFR Before and After Post-LKD Pregnancy

**FIGURE 3:**
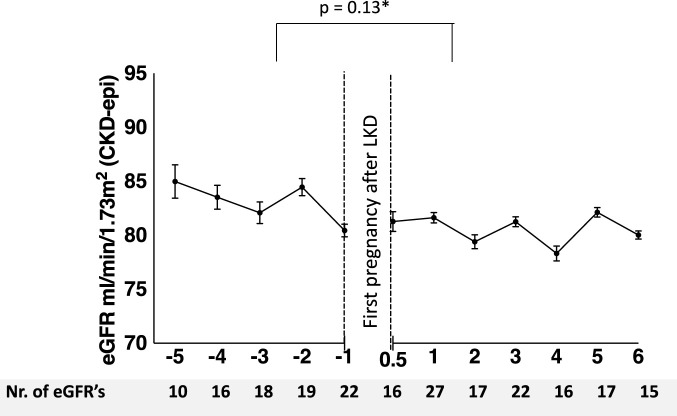
Adjusted mean eGFR before and after first pregnancy after living kidney donation (GEE multilevel analysis).

Furthermore, a sub-analysis of eGFR slope was performed solely in women with post-LKD pregnancies. Before pregnancy, 108 eGFR measurements were collected in 31 women, which results in a median of 4 measurements per women (IQR 3). After the first pregnancy, 275 eGFR measurements were collected in 37 women: 214 after a first post-LKD pregnancy, 49 after a second post-LKD pregnancy and 12 after a third post-LKD pregnancy. In total, a median of 7 measurements per woman (IQR 10) were included. eGFR analysis was adjusted for age at delivery, years after LKD and eGFR before LKD. The course of adjusted mean eGFR before and after first post-LKD delivery is illustrated in Figure 3. Adjusted mean eGFR before pregnancy was 78 mL/min/1.73 m^2^ (SEM 0.97, 95% CI 76.19–79.99) and after pregnancy 80 mL/min/1.73 m^2^ (SEM 0.60, 95% CI 78.50–80.84) (*p* = 0.13). eGFR slope per year was decreasing with −0.19 mL/min/1.73 m^2^ per year (SEM 0.42, 95% CI −1.01–0.62) before pregnancy, and after pregnancy with −0.23 mL/min/1.73 m^2^ per year (SEM 0.10, 95% CI −0.42; −0.43 (*p* < 0.001). Protein-creatinine ratio was not higher after pregnancy than before pregnancy (14 g/mol versus 15 g/mol, *p* = 0.83). Risk for a protein-creatinine ratio >15 g/mol was not significantly different after pregnancy compared to before pregnancy (OR 0.83, 95% CI 0.44–1.54, *p =* 0.55). MAP after pregnancy was not significantly different than before pregnancy (93 mmHg versus 89 mmHg, *p* = 0.09). Longer time between LKD and first delivery was associated with better eGFR (B 0.96, 95% CI 0.66–1.26, *p* < 0.001).

### Risk for Hypertension or Cardiovascular Events After Pregnancy in LKD

Long-term outcomes after LKD are demonstrated in Table 2. During our median follow-up time of 12 years after pregnancy, 14% of the women with only pre-LKD pregnancies used anti-hypertensive drugs versus 9% of the women with only post -KD pregnancies. Seven women were affected by a cardiovascular event (CVE) during follow-up. Six of these women had only pre-LKD pregnancies of whom one had a pre-LKD pregnancy with preeclampsia. She had a myocardial infarction 19 years after LKD. The seventh woman was nulliparous. Only two women developed diabetes mellitus type 2, one with only pre-LKD pregnancies and one was nulliparous. Due to the low incidence of these endpoints, we did not perform further statistical analysis.

**TABLE 2:** Characteristics during follow-up after Living kidney donation (LKD), *n* = 221, 2,149 visits.

	All	Only pregnant before LKD	Only pregnant after LKD	Pregnant before and after LKD	Woman after LKD with no pregnancies
Number of donors	221	137	23	18	43
Follow-up time after LKD (years) median {IQR}	12 (9)	12 (9)	15 (11)	10 (8)	10 (9)
Follow-up time after first pregnancy (years) median {IQR}	23 (12)	25 (12)	12 (12)	18 (11)	N/A
Nr. of eGFR measurements/donor median {IQR}	13 (10)	13 (11)	16 (7)	11 (7)	13 (11)
eGFR during follow-up (ml/min/1.73 m^2^) mean (±SD)	71 (12)	69 (12)	77 (10)	79 (11)	70 (15)
Hypertension during follow-up number (%)	122/221 (5)	77/137 (55)	13/23 (57)	7/18 (39)	25/43 (58)
Antihypertensive medication during follow-up number (%),	31/221 (14)	19/137 (14)	2/23 (9)	3/18 (17)	7/43 (16)
Proteinuria during follow-up* number (%)	135/221 (58)	87/137 (60)	13/23 (50)	8/18 (47)	27/43 (57)
Protein/creatinine ratio, mean (g/mol creatinine)** mean (±SD)	14 (10)	14 (9)	17 (18)	10 (4)	13 (10)
Cardiovascular events during follow-up number (%)	7/221 (3)	6/139 (4)	0	0	1/43 (2)

Hypertension was defined as systolic bloodpressure ≥140 mmHg and/or diastolic bloodpressure ≥90 mmHg and/or the use of anti-hypertensive drugs. eGFR (estimated glomerular filtration rate) was calculated using the CKD-epi formula. BMI: Body Mass Index, MAP: Mean Arterial Pressure Hypertension was defined as systolic blood pressure ≥140 mmHg and or a diastolic blood pressure ≥90 mmHg and or the use of antihypertensive medication. Proteinuria was defined as urine protein/creatinine ratio >15 g/mol creatinine, or urine albumin/creatinine ratio >3.5 mg/mmol or when no ratio could be calculated urine proteinuria >0.15 g/L. When none of these quantitative urine measurements were available a positive urine dipstick was defined as proteinuria. *17% missing data ** 66% missing data.

### Pregnancy Outcomes Before LKD Versus Pregnancy Outcomes After LKD

Pregnancy outcomes of pre-LKD pregnancies and post-LKD pregnancies are illustrated in Table 3. Miscarriages occurred in *n* = 23/84 (27%) in post-LKD pregnancies versus vs. 56/413 (14%) in pre-LKD pregnancies. Median year of miscarriage was 1996 (IQR 19) for pre-LKD miscarriages and 2012 (IQR 8) for post-LKD miscarriages. Univariable analysis demonstrated a higher risk for miscarriage in post-LKD pregnancies (OR 2.142, 95% CI 1.12–4.100, *p* = 0.021). Multivariable analysis adjusted for age and multiple pregnancies per women, showed no significant higher risk of miscarriages in post-LKD pregnancies versus pre-LKD pregnancies (OR 1.19, 95% CI 0.04–40.46, *p* = 0.92).

**TABLE 3:** Pregnancy outcomes before and after living kidney donation (LKD).

	Before LKD	After LKD
Number of donors with pregnancies	146	45
Number of pregnancies	413	86
Miscarriages, number (%)	56/413 (14)	23/86 (27)
Pregnancy duration at miscarriage (weeks)* median {IQR}	10 (6)	7 (4)
Abortion, number (%)	15 (4)	2 (2)
Pregnancies > 20 weeks		
Number of donors with pregnancies > 20 weeks	142	44
Number of pregnancies > 20 weeks	342	61
Primipara, number (%)	160/342 (47)	26/61 (43)
Follow-up time after first pregnancy (years), median {IQR}	24 (11)	12 (12)
Time between delivery and LKD (years), median {IQR}	−13 (10)	5 (4)
IUFD, number (%)	1 (<1)	1 (<1)
Pregnancy duration (weeks)**, mean (±SD)	39 (2)	39 (3)
Preterm birth with gestation of < 37 weeks, number (%)	31/321 (10)	8/61 (13)
Birthweight (Gram)***, mean (±SD)	3,493 (698)	3,254 (700)
Birthweight < 2,500 G, number (%)	16/324 (5)	3/59 (5)
Gestational hypertension, number (%)	22/341 (6)	8/61 (13)
Preeclampsia, number (%)	6/341 (2)	7/61 (11)
HELLP, number (%)	2/341 (<1)	0

IUFD: intra uterine fetal demise after 20 weeks of pregnancy, gestational hypertension: ≥140/90 mmHg measured twice after 20 weeks of pregnancy, Preeclampsia: presence of pregnancy induced hypertension >20 weeks of gestation and proteinuria. HELLP: Haemolysis Elevated Liver enzymes and Low Platelets Syndrome. 5 women were excluded of this analysis because of having only miscarriages or abortions. 1 woman with pre LKD abortion and post LKD miscarriage, 1 woman with only miscarriages post LKD and 3 women with only miscarriages before LKD. * 9/56 (16%) pre LKD miscarriages pregnancy duration missing, **21/342 (6%) pre LKD pregnancy duration missing, ***18/342 (5%) pre LKD pregnancies birthweight missing, 2/61 (3%) post LKD pregnancies birthweight missing.

Further analysis was performed only in women with pregnancies >20 weeks (*n* = 186 women, *n* = 402 pregnancies). Two women had an IUFD: one before LKD (at 33 weeks of pregnancy, unknown cause) and one after LKD (at 26 weeks of pregnancy, probably caused by a placenta infarction). According to the self-reported questionnaires 30/413 (7%) of the pregnancies pre-LKD were complicated by HDP and post-LKD 18/86 (21%) of the pregnancies were complicated by HDP. After studying their medical files, this alleged HDP could be confirmed in 15 pregnancies post-LKD: 7 with preeclampsia and 8 with gestational hypertension. Four pregnancies with preeclampsia had preterm birth and low birthweight. An overview of these women are shown in Supplementary Appendix SA.

In Table 4 we show the results of univariable and multivariable analyses on the risk of the composite outcome HDP, adjusted for multiple pregnancies in one woman. Post-LKD pregnancies had a significant higher risk of HDP, as well as higher MAP before LKD. In univariable and multivariable analysis, the risk of gestational hypertension was not higher in post-LKD pregnancies compared to pre-LKD pregnancies (OR 1.69, 95% CI 0.56–5.08, *p* = 0.35). However, post-LKD pregnancies did have a significantly higher risk of preeclampsia (OR 14.77, 95% CI 3.07–70.99, *p* = 0.001). Multivariable analysis identified that women with higher BMI at LKD (OR 1.26, 95% CI 1.08–1.46, *p* = 0.003) and lower parity (OR 0.38, 95% CI 0.18–0.76, *p* = 0.007) had a significantly higher risk of preeclampsia. All univariable analysis are presented in Supplementary Appendix SB.

**TABLE 4:** Multilevel risk of hypertensive disorders of pregnancy (HDP) (GEE multilevel logistic regression analysis) *n* = 186 women, 499 pregnancies.

	Univariable analysis			Multivariable analysis		
	Odds ratio	95% Confidence interval	*p*-value	Odds ratio	95% Confidence interval	*p*-value
Afro-American race	0.945	0.335–2.666	0.916			
Pregnancy after LKD	3.053	1.283–7.267	**0.012**	4.192	1.698–10.351	**0.002**
Gravida number	0.819	0.573–1.171	0.273			
Number of pregnancies> 20 weeks	0.595	0.385–0.922	**0.020**	0.539	0.344–0.845	**0.007**
BMI before LKD (kg/m^2^)	0.984	0.755–1.284	0.907			
MAP before LKD (mmHg)	1.068	1.011–1.128	**0.019**	1.066	1.032–1.102	**< 0.001**
Age at LKD (years)	0.919	0.777–1.087	0.324			
Year of LKD	1.084	0.956–1.184	0.258			
Age at delivery	0.998	0.931–1.071	0.959			
Year of delivery	1.030	0.928–1.145	0.589			

GEE: Generalized estimating equations, exchangeable matrix, Adjusted for multiple pregnancies in one woman. In bold the variables considered significant. Parity: the number of the pregnancy (>20 weeks), Gestational hypertension: ≥140/90 mmHg measured twice after 20 weeks of pregnancy. Hypertensive disorders of pregnancy: gestational hypertension, preeclampsia and/or HELLP, BMI: Body Mass Index, MAP: Mean arterial Pressure.

Multilevel univariable analysis on adverse pregnancy outcomes such as birthweight <2,500 g and preterm delivery were not associated with post-LKD pregnancies (*p* = 0.58 and *p* = 0.43) (Supplementary Appendix SC). In multilevel multivariable analysis preterm birth was associated with preeclampsia (OR 5.24, 95% CI 1.55–17.70, *p* = 0.008). Birthweight <2,500 g was associated with HDP during pregnancy (OR 4.875, 95% CI 1.607–14.790, *p* = 0.005). Absolute birthweight was significantly lower in pregnancies after LKD (*p* = 0.014, Supplementary Appendix SC).

## Discussion

To the best of our knowledge, this is the first study focusing on the effect of pregnancy on long-term kidney function and eGFR slope in pregnancies after LKD. Our main finding is that the eGFR slope after LKD is not different in women with or without pregnancy after LKD. Moreover, no difference in mean eGFR before and after post-LKD pregnancy was observed. Our second finding is that post-LKD pregnancies were more often complicated by HDP. However, no differences in adverse fetal outcomes were found when comparing pre- and post-LKD pregnancies. At last, we found that higher BMI and higher blood pressure at LKD were associated with adverse fetal and maternal outcomes during post-LKD pregnancy.

### Comparison With the Literature

We demonstrated for the first time that post-LKD pregnancy does not lead to a change in mean eGFR. Interestingly, we did find a small significant difference in eGFR slope before and after post-LKD pregnancy. eGFR decreased with −0.19 mL/min/1.73 m^2^ per year before pregnancy, and after pregnancy −0.23 mL/min/1.73 m^2^ per year (*p* < 0.001). This might be explained by the fact that eGFR slowly rises the first 4–5 years after LKD, and the median time between LKD and first delivery was 5 years (IQR 4). This phenomenon was recently described in two large prospective studies [17, 18]. In line with the aforementioned findings, we also found that longer time between LKD and first delivery was associated with better eGFR (*p* < 0.001).

Furthermore, none of the women with post-LKD pregnancies developed a CVE. No difference was found in the in incidence of hypertension in the group with only pre-LKD pregnancies compared to the group with only post-LKD pregnancies. It is known that women with a history of preeclampsia are more at risk for hypertension and CVEs later in life [19, 20]. Recent studies demonstrated that the risk of CVE is significantly lower after gestational hypertension and late onset preeclampsia compared to early onset preeclampsia [21, 22]. Most of the preeclampsia in our post-LKD pregnancies were late onset, mild preeclampsia and none of the women who were pregnant after LKD had a CVE. Of note is that these women had a rather short follow-up time (median 11 years after pregnancy) and in literature CVEs occur at longer periods of time after preeclampsia [23]. Women with HDP before LKD had a longer follow-up time (median 22 years) after the first pregnancy. They did not experience more CVEs or hypertension after LKD compared to women who did not experience HDP. We hypothesize that these women also had mild HDP as they still were able to pass the LKD screening and donated their kidney. In an earlier study in the general population, a similar non-negative effect of HDP was shown on kidney function after pregnancy [24].

In line with earlier literature, we also found a higher risk of HDP in post-LKD pregnancies [10]. It is important to discuss risks of post-LKD pregnancy with women considering pregnancy before LKD. We report an absolute risk for mild near-term preeclampsia of approximately 7% (versus 2%–4% in the general population). More-over we found a median of 200 g lower birth weight in post-LKD pregnancies (absolute, not corrected for confounders), but no increased risk for birth weight <2,500 g. Comparing outcomes of studies remains difficult, especially since studies use different definitions for gestational hypertension and preeclampsia. This higher risk of HDP in pregnancies post-LKD can also be explained by the higher age of the mothers who were pregnant after LKD [22]. Although in our study pregnancies post-LKD were more often complicated by HDP, in line with previous studies no differences were found in adverse fetal outcomes [10]. Furthermore, no difference was observed in eGFR after LKD with or without HDP, in line with an earlier study in the general population [24].

Higher BMI and blood pressure at LKD were associated with adverse pregnancy outcomes. As was shown earlier, women with a high BMI have smaller rest capacity after LKD and therefore are at higher risk for hypertension and HDP [25]. In studies in the general LKD population, hypertensive donors had no increased risk for reduced eGFR, proteinuria or ESRD compared to donors without hypertension [26]. However, whether obese donors have an increased risk of CVE or ESRD in comparison to the general LKD population has not fully been elucidated yet [27–29]. More research is warranted for counseling overweight women with a future pregnancy wish who want to donate their kidney.

### Strengths and Limitations

To date this is the first study that compared eGFR slopes after LKD in women with and without post—LKD pregnancies, and the first study that compared eGFR slopes before and after post-LKD pregnancy. Besides, we provide long-term follow-up data of pregnancies post-LKD up to 12 years after LKD, which has not been reported before. A few limitations should be taken into account, mainly due to the retrospective design of the study. There was some missing data and data on pregnancies was mainly self-reported, except for the complicated pregnancies after LKD. Furthermore, only a small group of women become pregnant after LKD. Due to these small numbers a prospective study on the effect of pregnancy on eGFR after LKD was not feasible. Therefore, the analysis to determine the impact of HDP on GFR decline and long-term CVE is probably not powered enough to detect small risks, but it does show that there is no large effect. There was a significant difference in age at LKD, eGFR before LKD, age at delivery, follow-up after delivery between the two groups, due to the study design. We adjusted for these differences in the model.

### Conclusion and Impact for Counseling

We conclude that pregnancy post-LKD does not affect kidney function of the mono-kidney at long-term follow-up, even when HDP occur. In line with existing literature, we report a higher risk of pregnancy complications, but this does not lead to an increase in adverse fetal outcomes. Therefore, our counseling advice is in line with British guidelines and KDIGO recommendations [30, 31]. We conclude that a pregnancy wish alone should not be a reason to exclude women for LKD. However, for women with high BMI or hypertension at the time of screening, a future pregnancy wish might be a reason to postpone or refrain from LKD.

## Data Availability

The data that support the findings of this study are available from the corresponding author upon reasonable request.

## Abbreviations

B, Coefficient estimate; BMI, Body mass index; CI, Confidence interval; CVE, cardiovascular event; eGFR, estimated Glomerular Filtration Rate; GEE, Generalized Estimating Equations; HDP, Hypertensive Disorders of Pregnancy; IUFD, Intra Uterine Fetal Demise; IQR, inter quartile range; LKD, Living Kidney Donation; MAP, mean arterial pressure; SCr, Serum creatinine; SD, Standard deviation; SEM, Standard error of the mean.
