# Supplementary material for: Long-Term Kidney and Maternal Outcomes After Pregnancy in Living Kidney Donors
Source: Transpl Int. 2023 Jun 28;36:11181. doi: 10.3389/ti.2023.11181 (PMC10337757; doi:10.3389/ti.2023.11181)
Supplement: Supplementary file 1 [file DataSheet1.docx]

**Appendix A: 15 women with hypertensive disorders of pregnancy**

|  | **Pre-existing comorbidities** | **Obstetric history** | **Gest. age** | **Birthweight** | **Mode of delivery** | **Type of hypertensive disorder of pregnancy** | **Anti-hypertensive drugs during pregnancy** | **Anti-hypertensive drugs at last visit** |
| --- | --- | --- | --- | --- | --- | --- | --- | --- |
| **A2** | Alport carrier | G3P1 | 40+2 | 3930 | Vaginal birth  Not induced | Gestational hypertension | No | Yes (ACE) |
| **B1** |  | G2P1 | 39+2 | 2890 | Vaginal birth  HT mother | Gestational hypertension  Preeclampsia | No | Yes (ARB) |
| **C1** | Factor V Leiden (Fraxiparine), DVT | G1P0 | 41+2 | 3370 | Vaginal birth  Not induced | Gestational hypertension | No | No |
| **D1** |  | G1P0 | 36+6 | 3260 | Vaginal birth  Not induced | Gestational hypertension | No | No |
| **D2** |  | G2P1 | 37+6 | 3305 | Vaginal birth  Dyspnoe, oedema | Gestational hypertension | Yes (methyldopa) | No |
| **E1** | BMI 42, hypertension proteinuria | G1P0 | 37+6 | 3355 | Vaginal birth  Fetal distress | Gestational hypertension  Preeclampsia | Yes (methyldopa) | Yes (CCB, BB, ARB) |
| **F1** | Factor V Leiden | G1P0 | 39+6 | 4770 | Vaginal birth  HT mother | Gestational hypertension | No | No |
| **G1** |  | G1P0 | 38 | 3500 | Vaginal birth  unknown | Gestational hypertension* | No | No |
| **H2** | Subfertility.  IVF pregnancy | G2P1 | 40+1 | 3040 | Vaginal birth  Not induced | Gestational hypertension | No | No |
| **I2** | Thrombocytopenia  Preeclampsia P1 | G5P1 | 38+3 | 2990 | Vaginal birth  Not induced | Gestational hypertension | No | Yes (CCB) |
| **J1** |  | G3P0 | 39+2 | 4320 | Vaginal birth  HT mother | Gestational hypertension  Preeclampsia | No | No |
| **L2** |  | G5P1 | 35+5 | 1995 | C-section  Fetal distress | Gestational hypertension  Preeclampsia | Yes (methyldopa) | Yes (ACE, methyldopa) |
| **M2** |  | G3P1 | 34 | 1605 | C-section  Fetal distress | Gestational hypertension  Preeclampsia | Yes (methyldopa) | No |
| **O2*** |  | G2P1 | 36 | 2500 | C-section  Fetal distress | Preeclampsia | Yes | Yes (ARB) |
| **P1** | Uterus myomatosus  IVF pregnancy | G5P0 | 31 | 1050 | C-section  Fetal distress | Gestational hypertension  Preeclampsia | Yes (methyldopa) | No |

**Appendix B: Adverse pregnancy outcomes, univariable analyses (GEE, multilevel)**

**Risk for preeclampsia (multilevel binary logistic regression, GEE)**

|  | **Univariable analysis** | | | **Multivariable analysis** | | |
| --- | --- | --- | --- | --- | --- | --- |
|  | **Odds Ratio** | **95% Confidence Interval** | **p- value** | **Odds Ratio** | **95% Confidence Interval** | **p- value** |
| Pregnancy after LKD | 10.715 | 2.933 - 39.151 | **0.000** | 14.773 | 3.074 – 70.985 | **0.001** |
| Pregnancy before LKD | * |  |  |  |  |  |
| Gravida number | 1.108 | 0.638 - 1.925 | 0.715 |  |  |  |
| Number of pregnancies > 20 weeks | 0.433 | 0.210 - 0.893 | **0.024** | 0.375 | 0.184 – 0.763 | **0.007** |
| Afro-american y/n | 1.893 | 0.484 - 7.359 | 0.359 |  |  |  |
| Age at delivery (years) | 1.063 | 0.953 - 1.186 | 0.273 |  |  |  |
| Age at LKD (years) | 0.936 | 0.838- 1.045 | 0.238 |  |  |  |
| BMI at LKD (kg/m^2^) | 1.206 | 1.105 - 1.316 | **0.000** | 1.256 | 1.078 – 1.463 | **0.003** |
| MAP before LKD (mmHg) | 1.023 | 0.978 – 1.070 | 0.323 |  |  |  |
| eGFR before LKD (ml/min/1.73m^2^) | 1.034 | 0.991 - 1.079 | 0.125 |  |  |  |
| Year of delivery | 1.059 | 0.974 - 1.152 | 0.182 |  |  |  |
| Year of LKD | 1.045 | 0.916 - 1.193 | 0.510 |  |  |  |

GEE: Generalized Estimated Equations (unstructured correlation matrix structure), LKD: Living Kidney Donation, BMI: Body Mass Index, MAP: Mean arterial Pressure, eGFR: estimated Glomerular Filtration Rate calculated by the CKD-EPI method, * Reference category

**Risk for gestational hypertension (multilevel binary logistic regression, GEE)**

|  | **Univariable analysis** | | | **Multivariable analysis** | | |
| --- | --- | --- | --- | --- | --- | --- |
|  | **Odds Ratio** | **95% Confidence Interval** | **p - value** | **Odds Ratio** | **95% Confidence Interval** | **p - value** |
| Pregnancy after LKD | 1.691 | 0.563 - 5.080 | 0.350 |  |  |  |
| Pregnancy before LKD | * |  |  |  |  |  |
| Gravida number | 0.644 | 0.394 - 1.053 | 0.079 |  |  |  |
| Number of pregnancies > 20 weeks | 0.526 | 0.319 - 0.867 | **0.012** | 0.570 | 0.331 – 0.982 | **0.043** |
| Afro-american (yes/no) | 0.593 | 0.120 - 2.893 | 0.518 |  |  |  |
| Age at delivery (years) | 0.969 | 0.883 - 1.065 | 0.516 |  |  |  |
| Age at LKD (years) | 0.886 | 0.742 - 1.058 | 0.181 |  |  |  |
| BMI at LKD (kg/m^2^) | 0.886 | 0.650 - 1.207 | 0.442 |  |  |  |
| MAP before LKD (mmHg) | 1.046 | 1.005 - 1.089 | **0.028** | 1.047 | 0.999 – 1.098 | 0.054 |
| eGFR before LKD (ml/min/1.73m^2^) | 1.044 | 0.983 - 1.109 | 0.164 |  |  |  |
| Year of delivery | 1.053 | 0.935 - 1.187 | 0.392 |  |  |  |
| Year of LKD | 1.106 | 1.039 - 1.177 | **0.002** | 0.921 | 0.847 – 1.001 | 0.053 |

GEE: Generalized Estimated Equations (unstructured correlation matrix structure), LKD: Living Kidney Donation, BMI: Body Mass Index, MAP: Mean arterial Pressure, eGFR: estimated Glomerular Filtration Rate calculated by the CKD-EPI method, * Reference category

**Appendix C: Adverse fetal outcomes, univariable and multivariable analyses (GEE, multilevel)**

**Risk for birthweight < 2500 gram (multilevel binary logistic regression, GEE)**

|  | **Univariable analysis** | | | **Multivariable analysis** | | |
| --- | --- | --- | --- | --- | --- | --- |
|  | **Odds Ratio** | **95% Confidence Interval** | **p value** | **Odds Ratio** | **95% Confidence Interval** | **p value** |
| Pregnancy after LKD | 1.351 | 0.472 -3.869 | 0.575 |  |  |  |
| Pregnancy before LKD | * |  |  |  |  |  |
| Gravida number | 1.004 | 0.667 - 1.510 | 0.986 |  |  |  |
| Number of pregnancy > 20 weeks | 3.741 | 0.869 - 16.102 | 0.076 |  |  |  |
| Afro-American yes/no | 0.608 | 0.214 - 1.731 | 0.352 |  |  |  |
| Age at delivery (years) | 0.945 | 0.848 - 1.053 | 0.308 |  |  |  |
| Age at LKD (years) | 1.000 | 0.916 - 1.091 | 0.996 |  |  |  |
| BMI at LKD (kg/m^2^) | 1.053 | 0.953 - 1.164 | 0.309 |  |  |  |
| MAP before LKD (mmHg) | 1.036 | 1.011 - 1.061 | **0.004** | 1.018 | 0.994 – 1.043 | 0.136 |
| eGFR before LKD (ml/min/1.73m^2^) | 0.975 | 0.955 - 0.995 | **0.014** | 1.020 | 0.994 – 1.047 | 0.137 |
| Year of delivery | 0.984 | 0.935 - 1.036 | 0.537 |  |  |  |
| Year of LKD | 0.963 | 0.933 - 1.068 | 0.998 |  |  |  |
| Hypertensive disorder of pregnancy yes/no | 7.005 | 2.680 - 18.312 | **0.000** | 4.875 | 1.607 – 14.790 | **0.005** |
| Gestational hypertension yes/no | 3.627 | 1.110 - 11.845 | **0.033** |  |  |  |
| Preeclampsia yes/no | 6.762 | 1.732 - 26.402 | **0.006** |  |  |  |

GEE: Generalized Estimated Equations (unstructured correlation matrix structure), LKD: Living Kidney Donation, BMI: Body Mass Index, MAP: Mean arterial Pressure, eGFR: estimated Glomerular Filtration Rate calculated by the CKD-EPI method, * Reference category

**Risk for preterm birth < 37 weeks (multilevel binary logistic regression, GEE)**

|  | **Odds Ratio** | **95% Confidence Interval** | **p value** |
| --- | --- | --- | --- |
| Pregnancy after LKD | 1.389 | 0.610 - 3.164 | 0.434 |
| Pregnancy before LKD | * |  |  |
| Number of pregnancies > 20 weeks | 2.245 | 0.766 - 6.580 | 0.140 |
| Gravida number | 1.111 | 0.713 - 12.952 | 0.133 |
| Afro-American | 1.679 | 0.776 – 3.633 | 0.188 |
| Age at delivery (years) | 1.096 | 0.938 - 1.280 | 0.249 |
| Age at LKD (years) | 1.016 | 0.809 - 1.275 | 0.893 |
| BMI at LKD (kg/m^2^) | 1.230 | 0.957 - 1.580 | 0.106 |
| MAP before LKD (mmHg) | 1.056 | 0.940 - 1.186 | 0.358 |
| eGFR before LKD (ml/min/1.73m^2^) | 0.995 | 0.947 - 1.046 | 0.857 |
| Year of delivery | 1.053 | 0.979 - 1.134 | 0.166 |
| Year of LKD | 0.947 | 0.718 - 1.247 | 0.697 |
| **Hypertensive disorder of pregnancy yes/no** | **2.647** | **1.294 - 5.415** | **0.008** |
| Gestational hypertension yes/no | 1.013 | 0.375 - 2.733 | 0.980 |
| **Preeclampsia yes/no** | **5.240** | **1.551 - 17.696** | **0.008** |

GEE: Generalized Estimated Equations (unstructured correlation matrix structure), LKD: Living Kidney Donation, BMI: Body Mass Index, MAP: Mean arterial Pressure, eGFR: estimated Glomerular Filtration Rate calculated by the CKD-EPI method, * Reference category

**Risk for lower birthweight (multilevel linear regression model, GEE)**

|  | **Univariable analysis** | | | **Multivariable analysis** | | |
| --- | --- | --- | --- | --- | --- | --- |
|  | **B** | **95% Confidence Interval** | **Sig.** | **B** | **95% Confidence Interval** | **p value** |
| **Pregnancy after LKD** | **-208.875** | **-376.027 - -41.722** | **0.014** | **-232.039** | **-463.449 - -0.629** | **0.049** |
| Pregnancy before LKD | * |  |  |  |  |  |
| Number of pregnancies > 20 weeks | 14.112 | -37.732 – 65.955 | 0.594 |  |  |  |
| Gravida number | 10.908 | -41.380 – 63.196 | 0.683 |  |  |  |
| Afro-American yes/no | -165.933 | -464.791 – 132.924 | 0.276 |  |  |  |
| Age at delivery | 1.992 | -10.054 – 14.038 | 0.746 |  |  |  |
| Age at LKD | 8.536 | -5.092 – 22.163 | 0.220 |  |  |  |
| BMI at LKD | 1.569 | -18.135 – 21.274 | 0.876 |  |  |  |
| MAP before LKD | 1.536 | -8.754 – 11.826 | 0.770 |  |  |  |
| eGFR before LKD | -2.163 | -7.259 - 2.934 | 0.406 |  |  |  |
| Year of delivery | -2.583 | -10.813 – 5.646 | 0.538 |  |  |  |
| Year of donation | -4.720 | -16.903 – 7.464 | 0.448 |  |  |  |
| **Hypertensive disorder of pregnancy yes/no** | **-258.051** | **-498.862 - -17.241** | **0.036** | **-181.190** | **-333.379 - -29.002** | **0.020** |
| Gestational hypertension yes/no | -91.309 | -346.083 - 163.466 | 0.493 |  |  |  |
| Preeclampsia yes/no | -445.493 | -989.723 - 98.736 | 0.109 |  |  |  |

GEE: Generalized Estimated Equations (unstructured correlation matrix structure), LKD: Living Kidney Donation, BMI: Body Mass Index, MAP: Mean arterial Pressure, eGFR: estimated Glomerular Filtration Rate calculated by the CKD-EPI method, * Reference category
